# Supplementary material for: A continuum of zinc finger transcription factor retention on native chromatin underlies dynamic genome organization
Source: Mol Syst Biol. 2024 May 14;20(7):4. doi: 10.1038/s44320-024-00038-5 (PMC11220090; doi:10.1038/s44320-024-00038-5)
Supplement: Supplementary file 1 — Appendix [file 44320_2024_38_MOESM1_ESM.pdf]

Appendix

A continuum of zinc finger transcription factor retention on native chromatin underlies dynamic genome organization

Siling Hu[1,2,3,4], Yangying Liu[1,2,3,4], Qifan Zhang[1,2,3], Juan Bai[1,2,3], Chenhuan Xu[1,2,3]*

[1]CAS Key Laboratory of Genome Sciences and Information, Beijing Institute of Genomics, Chinese Academy of Sciences, Beijing 100101, China. [2]China National Center for Bioinformation, Beijing 100101, China. [3]University of Chinese Academy of Sciences, Beijing 100049, China. [4]These authors contributed equally: Siling Hu, Yangying Liu. *E-mail: xuchh@big.ac.cn

TABLE OF CONTENTS

Appendix Figure S1. Native chromatin-retained CTCF sites exhibit functional conservation. 2

Appendix Figure S2. TFs display differential susceptibility to zinc depletion on native chromatin. 3

Appendix Figure S3. The continuum of CTCF retention translates into concordant stability of chromatin structures under zinc depletion. 4

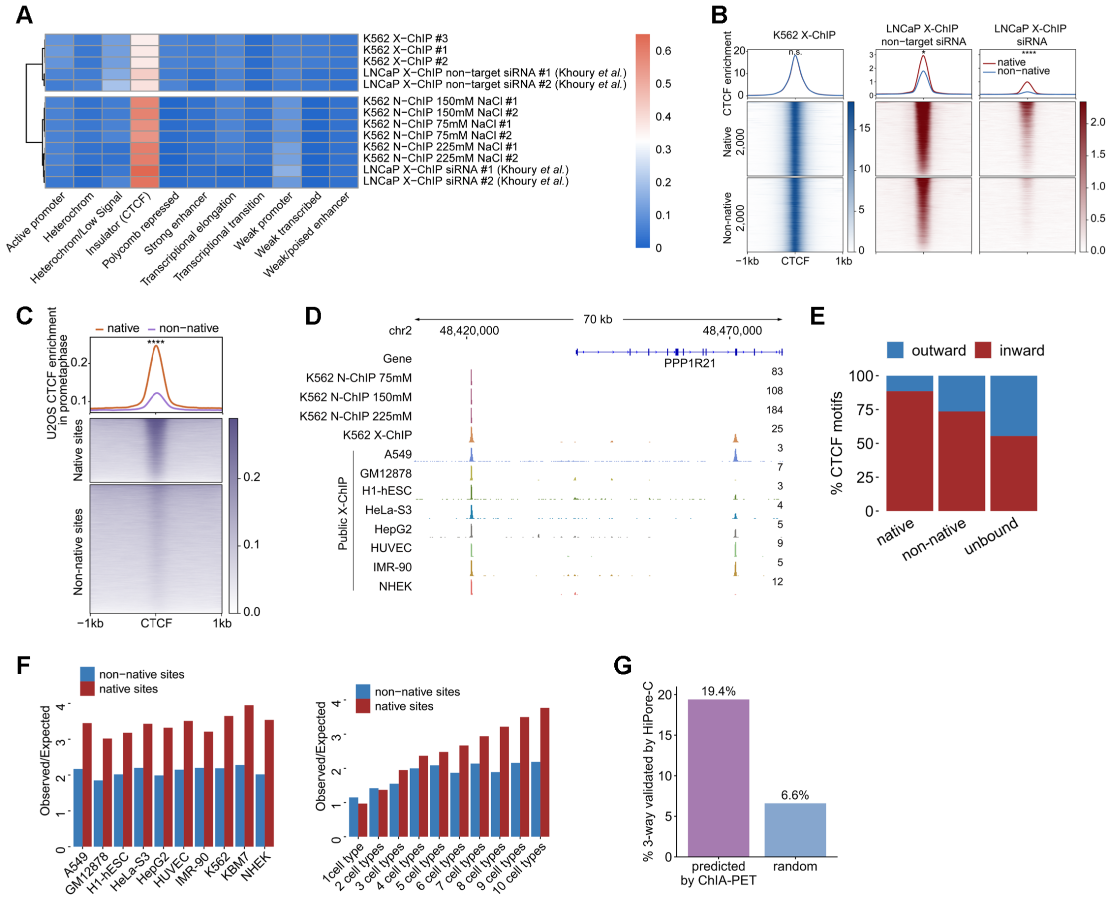

Appendix Figure S1. Native chromatin-retained CTCF sites exhibit functional conservation.

(**A**) Hierarchically clustered heatmap showing the chromatin state distribution for each peak set annotated by K562 ChromHMM chromatin states. (**B**) Average profiles and heatmaps representing normalized K562 CTCF X-ChIP signals, and LNCaP CTCF X-ChIP signals under control or siCTCF conditions, centered around CTCF motifs. Each 2000 CTCF motifs are selected from both native and non-native motif groups with equal K562 X-ChIP signals. n.s., not significant, $*P < 0.05$, $****P < 0.0001$; two-tailed *t*-test. (**C**) Average profile and heatmaps showing the distribution of U2OS CTCF signals during prometaphase at native and non-native sites. $****P < 0.0001$, two-tailed *t*-test. (**D**) An example showing that native sites are constitutively bound by CTCF in different cell lines. (**E**) The percentage of native, non-native or unbound CTCF motifs within Hi-C loop anchors that face inward or outward towards the loop domains. (**F**) The observed/expected ratio for non-native and native sites overlapping with domain boundaries of distinct cell types (left) and domain boundary subsets as in Fig. 5E (right). (**G**) Percentage of predicted or random 3-way ChIA-PET contacts validated by HiPore-C data.

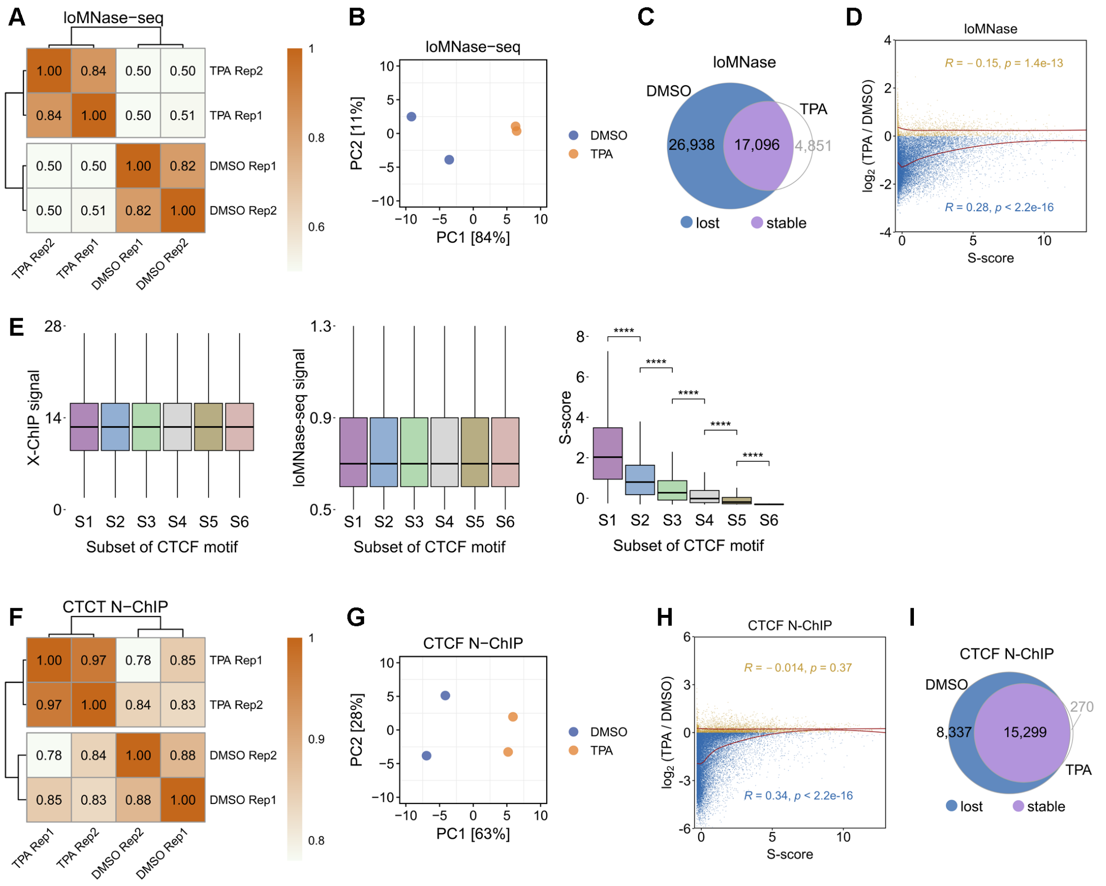

Appendix Figure S2. TFs display differential susceptibility to zinc depletion on native chromatin.

(**A**) Heatmap showing Pearson correlation coefficients between loMNase-seq biological replicates under DMSO or TPA conditions. (**B**) Principal component analysis of loMNase-seq biological replicates under DMSO or TPA conditions. (**C**) Venn diagram showing number of lost, stable, or gained loMNase-seq peaks between DMSO and TPA conditions. (**D**) Scatter plot showing the correlations between $\log_2$(TPA/DMSO) values of loMNase-seq and S-score. The loess-fitted lines represent the trends. Dots represent CTCF motifs. Pearson correlation and the two-sided significance level are shown. (**E**) The boxplots showing the X-ChIP, loMNase-seq signals and S-score of the six groups of CTCF motifs. The six groups of CTCF motifs are grouped based on identical X-ChIP and loMNase signals (see Materials and Methods). For each subset, the number of CTCF motifs is 1,637. The central band represents the median. The lower and upper hinges represent the first and third quartiles, respectively. The whiskers represent the 1.5 × interquartile range. ****$P < 0.0001$; two-sided Wilcoxon rank sum test. (**F**) Heatmap showing Pearson correlation coefficients between CTCF N-ChIP biological replicates under DMSO or TPA conditions. (**G**) Principal component analysis of CTCF N-ChIP biological replicates under DMSO or TPA conditions. (**H**) Scatter plot showing the correlations between $\log_2$(TPA/DMSO) values of CTCF N-ChIP and S-score. The loess-fitted lines represent the trends. Dots represent CTCF motifs. Pearson correlation and the two-sided significance level are shown. (**I**) Venn diagram showing number of lost, stable, or gained CTCF N-ChIP peaks between DMSO and TPA conditions.

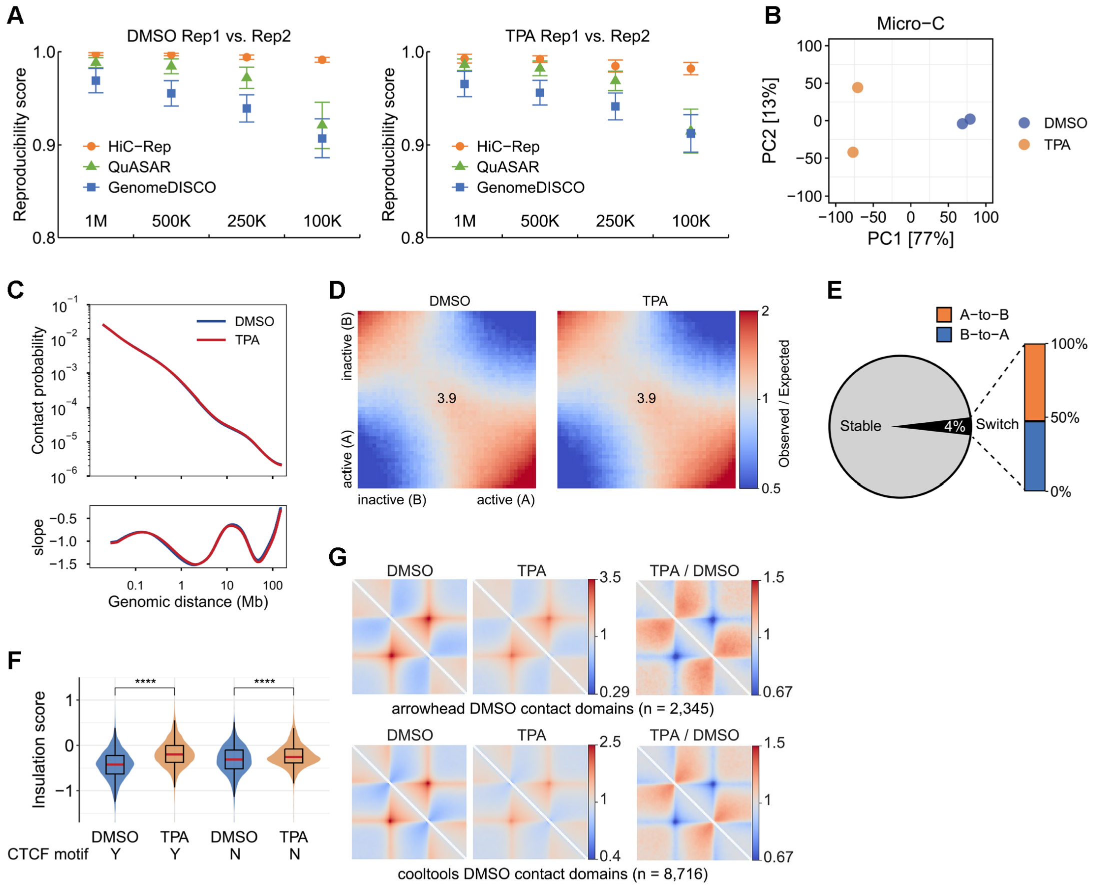

Appendix Figure S3. The continuum of CTCF retention translates into concordant stability of chromatin structures under zinc depletion.

(**A**) The reproducibility scores (Y axis) of Micro-C biological replicates at different resolutions (X axis) using three methods. The reproducibility scores were individually calculated for chr1 to chr22 (n = 22). Error bars represent the mean ± standard deviation. (**B**) Principal component analysis of Micro-C biological replicates under DMSO or TPA conditions. (**C**) Genome-wide contact decaying *P(s)* analysis (top) and slope distribution of the *P(s)* curves (bottom) for Micro-C data from the DMSO- or TPA-treated cells. (**D**) Saddle plots showing the compartmentalization in the DMSO- or TPA-treated cells. The regions are sorted by PC1 value from B to A compartment and interactions between different regions are shown in the heatmap. The numbers indicate the compartment strength. (**E**) 100-kb genomic bins that switch compartment (4% of the genome) due to TPA treatment. Bar indicates the distribution of A-to-B and B-to-A switches. (**F**) Violin plot and boxplot of insulation scores of boundaries containing CTCF motifs (Y) or not (N) under DMSO or TPA conditions. Number of boundaries (from left to right): 7,368 / 7,368 / 6,711 / 6,711. For boxplot, the red central band represents the median. The lower and upper hinges represent the first and third quartiles, respectively. The whiskers represent the 1.5 × interquartile range. ****$P < 0.0001$; two-sided t-test. (**G**) Heatmaps of the average observed/expected Micro-C interactions in the contact domains under DMSO or TPA conditions.
