# Supplementary material for: A continuum of zinc finger transcription factor retention on native chromatin underlies dynamic genome organization
Source: Mol Syst Biol. 2024 May 14;20(7):4. doi: 10.1038/s44320-024-00038-5 (PMC11220090; doi:10.1038/s44320-024-00038-5)
Supplement: Supplementary file 4 — Expanded View Figures [file 44320_2024_38_MOESM4_ESM.pdf]

Expanded View Figures

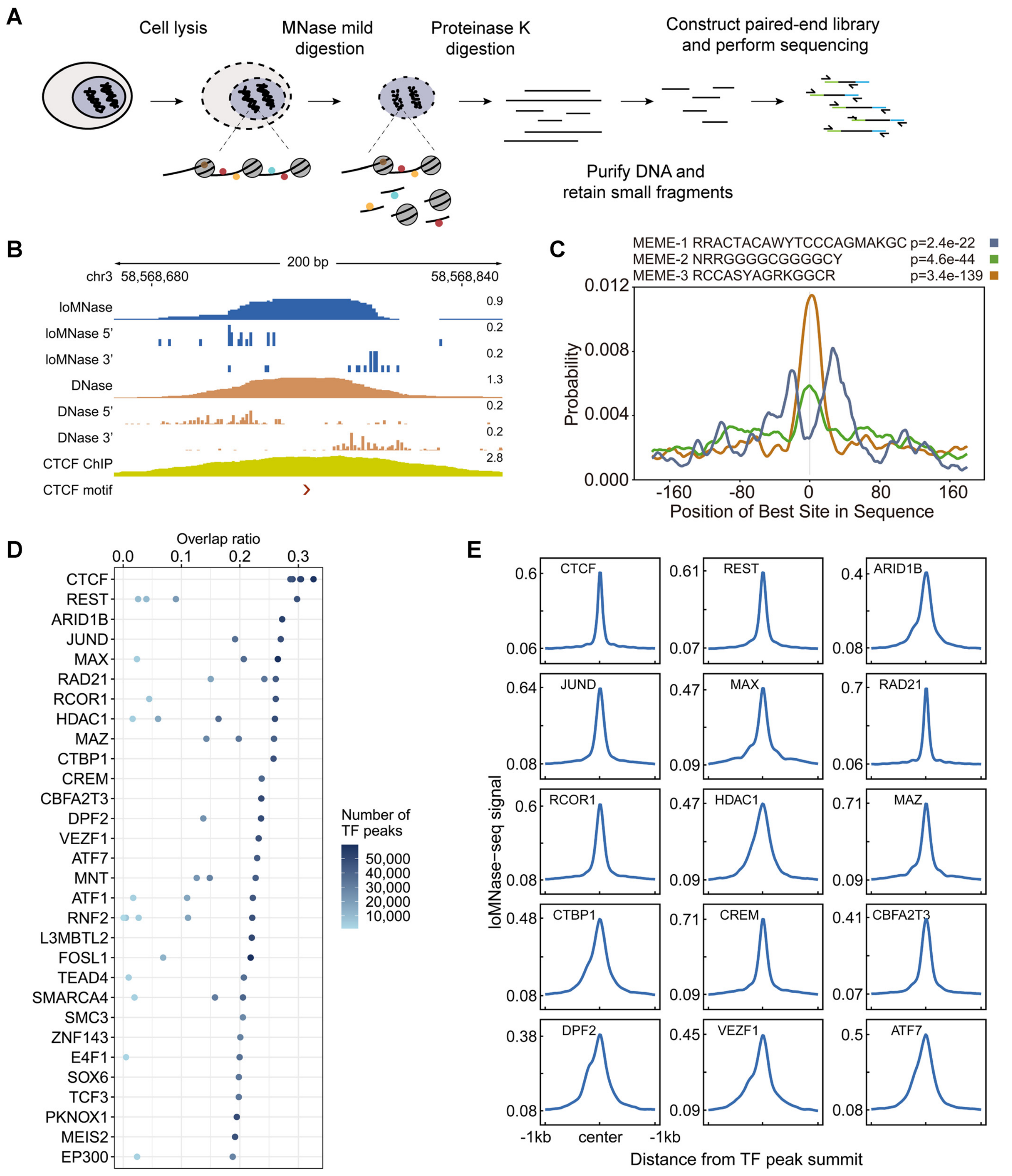

 © The Author(s)

Figure EV1. loMNase-seq maps high-resolution TF footprints on native chromatin.

(**A**) Schematic workflow of loMNase-seq. (**B**) Genome browser representation of normalized reads for loMNase-seq, DNase-seq, and CTCF ChIP-seq, as well as the fragment endpoints of loMNase-seq and DNase-seq relative to an example CTCF motif. CTCF motif orientation is indicated by a red arrow. (**C**) The CentriMo plot showing the distribution of the three most optimal MEME-ChIP-derived motifs in the top 3000 loMNase-seq peak regions. The centrally positioned vertical line corresponds to the midpoint of the sequences. MEME-1, -2, -3 correspond to Motif 1, 2, 3 respectively shown in Fig. 1E. The *P* value derived from CentriMo tool represents the statistical significance of the enrichment of the motif, adjusted for multiple tests. The *P* value is calculated by one-tailed binomial test. (**D**) The top 30 TFs were ranked based on their maximum overlap ratio with loMNase-seq peaks. Each dot represents a ChIP-seq result for a specific TF obtained from ENCODE database. (**E**) Enrichment of loMNase-seq signals at different TF ChIP-seq peak sets.

© The Author(s)

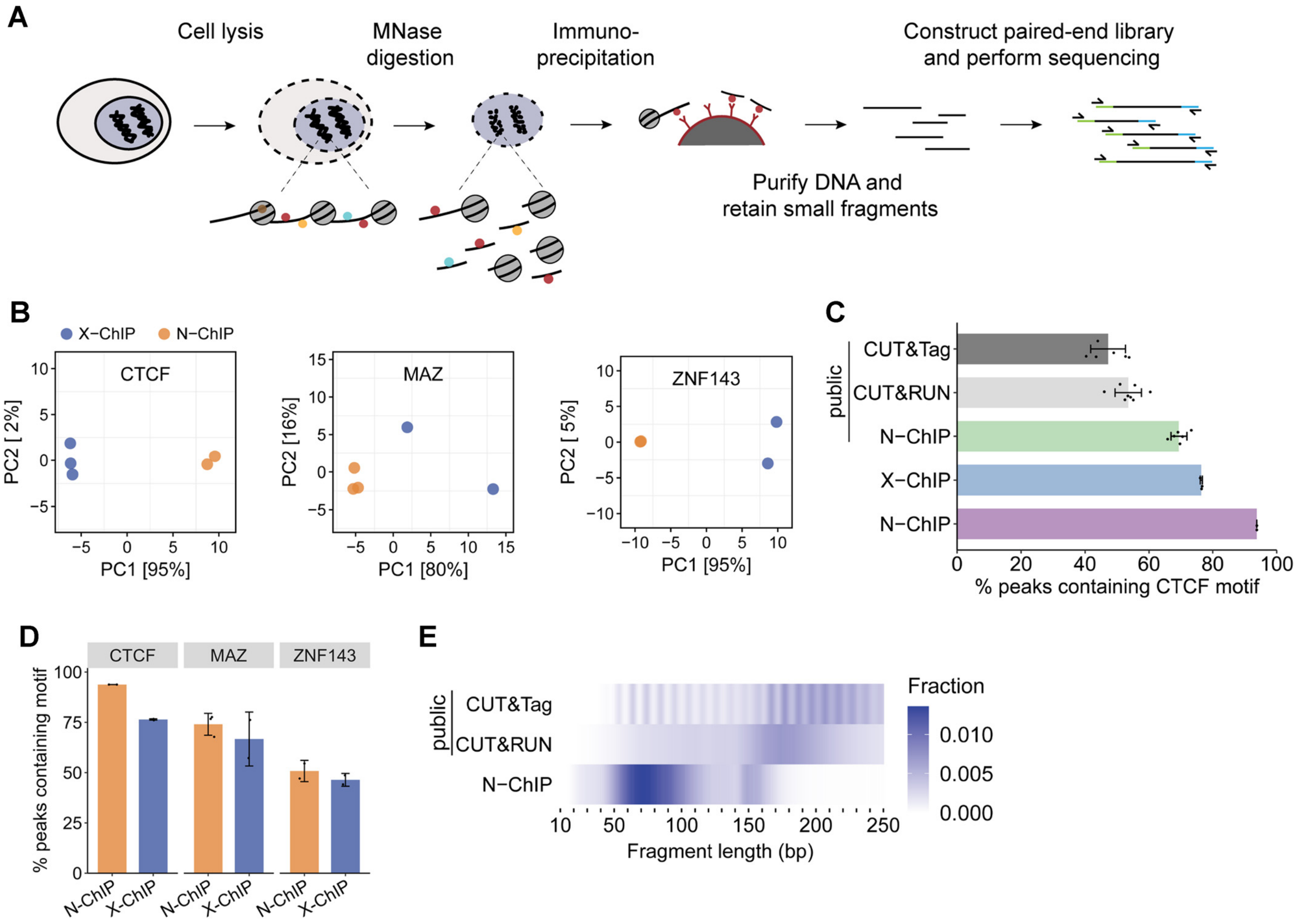

Figure EV2. Occupancy of single TFs on native chromatin.

(**A**) Schematic workflow of N-ChIP. (**B**) Principal component analysis (PCA) of N-ChIP and X-ChIP datasets for CTCF, MAZ or ZNF143. Each dot represents an individual replicate. (**C**) Percentage of peaks containing CTCF canonical motifs for previously published CUT&Tag, CUT&RUN, N-ChIP, as well as N-ChIP and X-ChIP datasets generated in this study. Each dot represents an individual experiment or replicate. Numbers of samples (from top to bottom): $n = 6/8/6/6/3/2$. Error bars represent the mean ± standard deviation. (**D**) Percentage of CTCF, MAZ or ZNF143 peaks containing their respective motifs for N-ChIP or X-ChIP datasets. Numbers of samples (from left to right): $n = 2/3/3/2/2/2$. Error bars represent the mean ± standard deviation. (**E**) Fragment length distribution of CUT&Tag, CUT&RUN or N-ChIP datasets.

 © The Author(s)

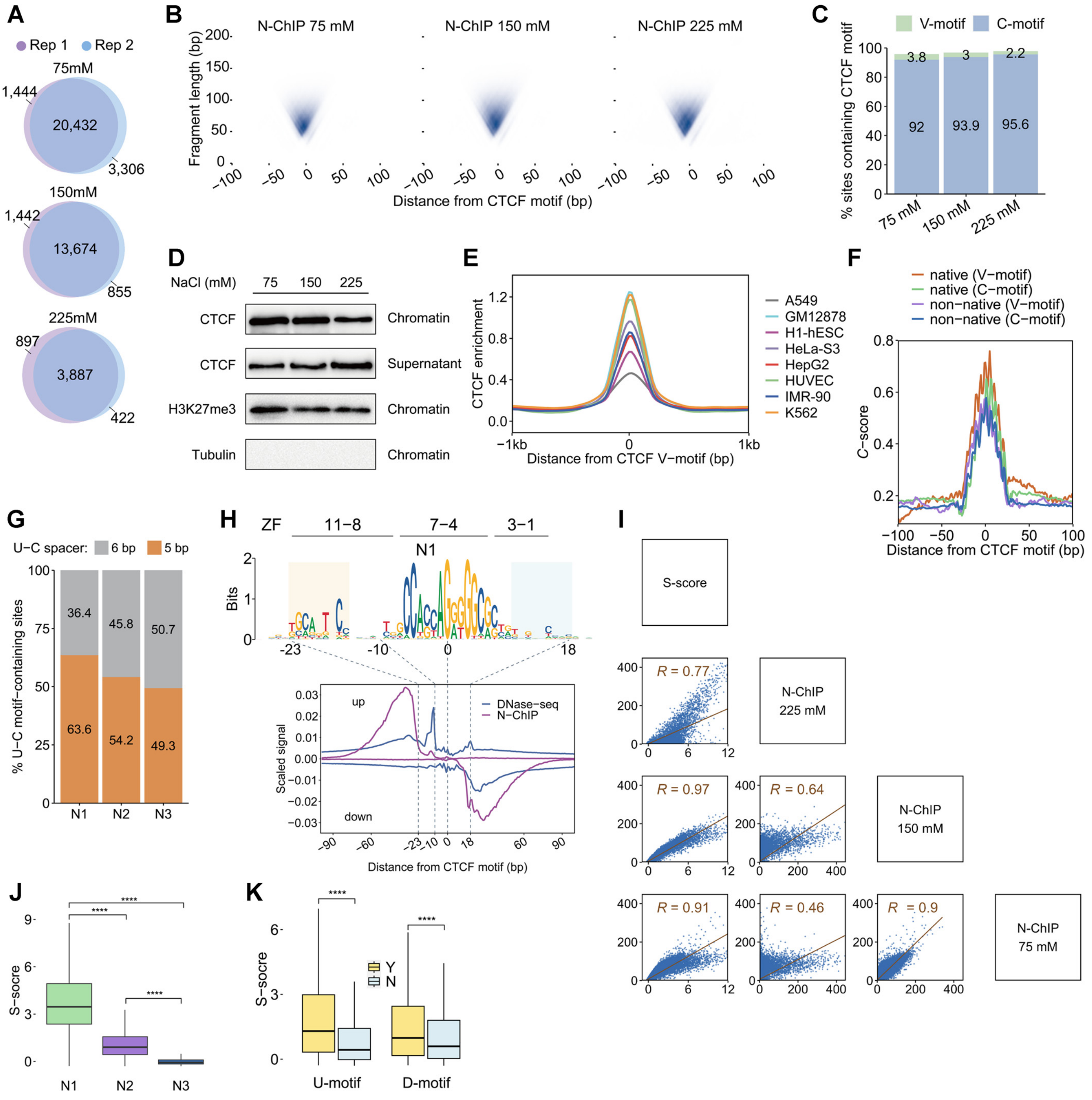

Figure EV3. A continuum of CTCF retention across genomic sites on native chromatin.

(**A**) Venn diagrams showing reproducibility between biological replicates of CTCF N-ChIP under 75, 150, or 225 mM NaCl concentration. (**B**) V-plots of 75, 150, or 225 mM CTCF N-ChIP fragments at CTCF motifs. (**C**) Percentage of 75, 150, or 225 mM CTCF N-ChIP peaks containing C- or V-motifs. (**D**) Western blot of extracted chromatin fractions. The chromatin and supernatant on right side mark the insoluble and soluble fractions respectively, after incubating the extracted chromatin fraction in 75, 150, or 225 mM NaCl. H3K27me3 and Tubulin serve as controls for chromatin and cytoplasm respectively. (**E**) The normalized CTCF X-ChIP signal of eight different cell types at CTCF V-motifs. (**F**) Mean predicted cyclizability score (*C*-score) around C- or V-motifs present in native or non-native sites. (**G**) The percentage of CTCF motifs with different U-C spacer length from N1, N2 and N3 groups. (**H**) The scaled pileup of fragment 5′ and 3′ ends around CTCF motifs. Motif logo is shown at the top. The ZF domains binding to these regions are indicated. (**I**) Pairwise scatterplots showing the Pearson correlation between S-score and normalized N-ChIP signals. (**J**) Boxplot showing the S-score of CTCF motifs within N1, N2 and N3 sites (N1, $n = 3669$; N2, $n = 8823$; N3, $n = 6304$). ****$P < 0.0001$; two-sided Wilcoxon rank sum test. (**K**) Comparison of S-score of N1, N2 and N3 C-motifs with (Y) or without (N) auxiliary motifs. Numbers of C-motifs (from left to right): $n = 5632/13{,}164/2032/16{,}764$). ****$P < 0.0001$; two-sided Wilcoxon rank sum test. Data information: For boxplots in (**J**, **K**), the central band represents the median. The lower and upper hinges represent the first and third quartiles, respectively. The whiskers represent the 1.5× interquartile range.

© The Author(s)

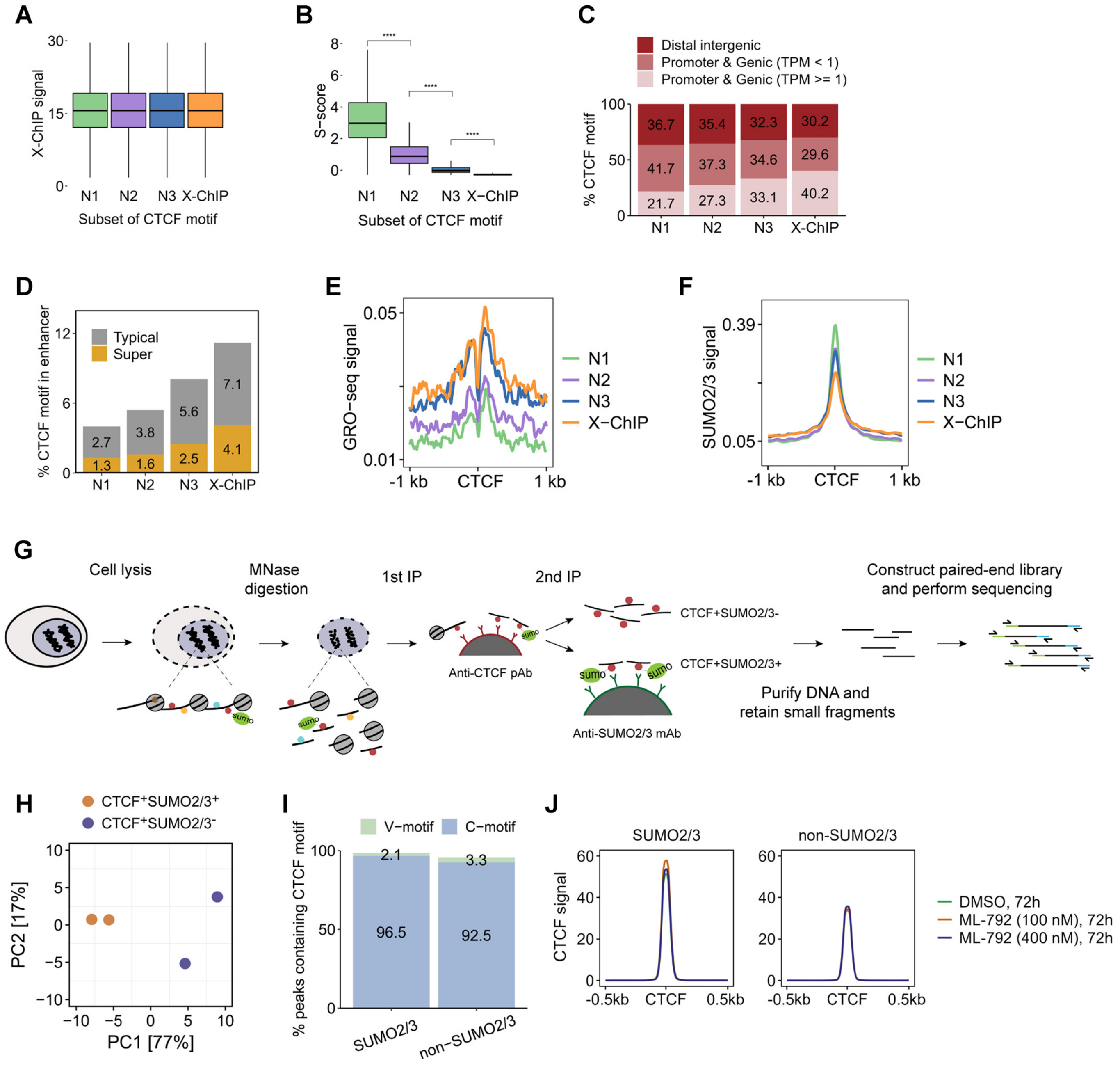

Figure EV4. CTCF retention on native chromatin associates with transcriptional activity and post-translational modifications.

(**A**) Boxplot showing the X-ChIP signals of sampled CTCF motifs from N1, N2, N3 and X-ChIP groups. For each subset, the number of CTCF motifs is 1,939. (**B**) Boxplot showing the S-score of sampled CTCF motifs from N1, N2, N3 and X-ChIP groups. For each subset, the number of CTCF motifs is 1,939. ****$P < 0.0001$; two-sided Wilcoxon rank sum test. (**C**) Percentage of sampled CTCF motifs from N1, N2, N3 and X-ChIP groups located within distal intergenic, or promoter and genic (TPM < 1 or ≥ 1) regions. (**D**) Percentage of sampled CTCF motifs from N1, N2, N3 and X-ChIP groups located within typical or super enhancers. (**E**) GRO-seq signals at sampled CTCF motifs from N1, N2, N3 and X-ChIP groups. (**F**) SUMO2/3 X-ChIP signal at sampled CTCF motifs from N1, N2, N3 and X-ChIP groups. (**G**) Schematic workflow of sequential N-ChIP. (**H**) PCA of CTCF-SUMO2/3 sequential N-ChIP datasets. Each dot represents an individual replicate. (**I**) Percentage of SUMO2/3 and non-SUMO2/3 sites containing C- or V-motifs. (**J**) Enrichment of CTCF N-ChIP signals around CTCF motifs within SUMO2/3 and non-SUMO2/3 sites under SUMOylation inhibition. Data information: For boxplots in (**A**, **B**), the central band represents the median. The lower and upper hinges represent the first and third quartiles, respectively. The whiskers represent the 1.5× interquartile range.

 © The Author(s)

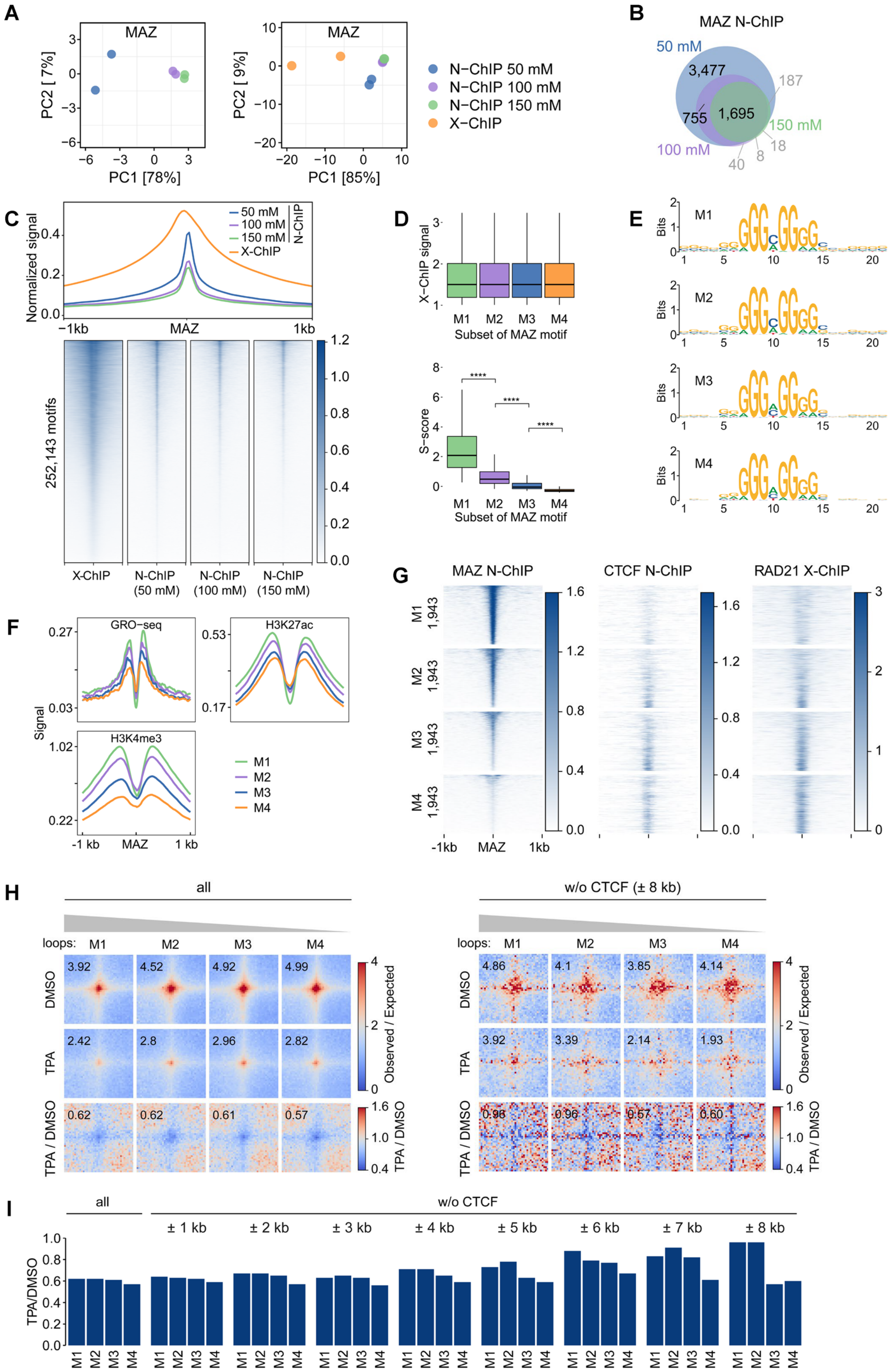
A
MAZ
PC1 [78%]
PC2 [7%]
PC1 [85%]
PC2 [9%]
N-ChIP 50 mM
N-ChIP 100 mM
N-ChIP 150 mM
X-ChIP
B
MAZ N-ChIP
50 mM
3,477
187
755
1,695
150 mM
100 mM
18
8
40
C
Normalized signal
50 mM
100 mM
150 mM
N-ChIP
X-ChIP
−1kb
MAZ
1kb
252,143 motifs
X-ChIP
N-ChIP (50 mM)
N-ChIP (100 mM)
N-ChIP (150 mM)
D
X-ChIP signal
Subset of MAZ motif
S-score
E
M1
M2
M3
M4
Bits
F
GRO-seq
H3K27ac
H3K4me3
Signal
-1 kb
MAZ
1 kb
G
MAZ N-ChIP
CTCF N-ChIP
RAD21 X-ChIP
M1 1,943
M2 1,943
M3 1,943
M4 1,943
H
all
w/o CTCF (± 8 kb)
loops:
DMSO
TPA
TPA / DMSO
Observed / Expected
I
all
w/o CTCF
± 1 kb
± 2 kb
± 3 kb
± 4 kb
± 5 kb
± 6 kb
± 7 kb
± 8 kb
TPA/DMSO

Figure EV5. The S-score delineates MAZ retention on native chromatin and stability of MAZ-mediated chromatin loops independent of CTCF.

(**A**) Principal component analysis of MAZ N-ChIP datasets with (right) or without (left) X-ChIP datasets. Each dot represents an individual replicate. (**B**) Venn diagram showing the overlap between 50, 100, and 150 mM MAZ N-ChIP peaks. (**C**) Average profiles and heatmaps showing normalized MAZ X-ChIP and N-ChIP signals centered around the MAZ motifs. (**D**) The boxplots showing the X-ChIP signals and S-score of four groups of MAZ motifs. The four groups of MAZ motifs are grouped based on identical X-ChIP signals (see "Methods"). For each subset, the number of MAZ motifs is 1943. The central band represents the median. The lower and upper hinges represent the first and third quartiles, respectively. The whiskers represent the 1.5× interquartile range. ****$P < 0.0001$; two-sided Wilcoxon rank sum test. (**E**) Sequence logo representation for the four groups of MAZ motifs as described in (**D**). (**F**) Average profiles showing GRO-seq signals and ChIP signals of different histone modifications centered around the four groups of MAZ motifs as described in (**D**). (**G**) Heatmaps showing MAZ N-ChIP (50 mM), CTCF N-ChIP (75 mM) and RAD21 X-ChIP signals centered around the four groups of MAZ motifs as described in (**D**). (**H**) APA plots showing the changes of chromatin loop strength under TPA treatment. The loops are grouped based on the presence of MAZ motifs from the four groups at their anchors, as defined in (**D**). Right panel: same as left panel but Micro-C pairs with CTCF motifs within ±8 kb were excluded to diminish contribution by CTCF-mediated loops. Bin size: 5 kb. (**I**) Barplot showing the fold change of chromatin loop strength between the TPA and DMSO conditions. Micro-C pairs with CTCF motifs within ±1, ±2, ±3, ±4, ±5, ±6, ±7 or ±8 kb were consecutively excluded to diminish the contribution by CTCF-mediated loops.

 © The Author(s)
